# IL-12Rβ1 Deficiency in Two of Fifty Children with Severe Tuberculosis from Iran, Morocco, and Turkey

**DOI:** 10.1371/journal.pone.0018524

**Published:** 2011-04-13

**Authors:** Stéphanie Boisson-Dupuis, Jamila El Baghdadi, Nima Parvaneh, Aziz Bousfiha, Jacinta Bustamante, Jacqueline Feinberg, Arina Samarina, Audrey V. Grant, Lucile Janniere, Naima El Hafidi, Amal Hassani, Daniel Nolan, Jilali Najib, Yildiz Camcioglu, Nevin Hatipoglu, Cigdem Aydogmus, Gonul Tanir, Caner Aytekin, Melike Keser, Ayper Somer, Guside Aksu, Necil Kutukculer, Davood Mansouri, Alireza Mahdaviani, Setareh Mamishi, Alexandre Alcais, Laurent Abel, Jean-Laurent Casanova

**Affiliations:** 1 St. Giles Laboratory of Human Genetics of Infectious Diseases, Rockefeller Branch, The Rockefeller University, New York, New York, United States of America; 2 Laboratory of Human Genetics of Infectious Diseases, U980, Institut National de la Santé et de la Recherche Médicale (INSERM), Paris, France; 3 Necker Medical School, University Paris Descartes, Paris, France; 4 Genetics Unit, Military Hospital Mohamed V, Hay Riad Rabat, Morocco; 5 Department of Pediatrics, Infectious Disease Research Center, Teheran University of Medical Sciences, Teheran, Iran; 6 Clinical Immunology Unit, King Hassan II University, Ibn-Rochd Hospital, Casablanca, Morocco; 7 Department of Pediatrics, Rabat Children Hospital, Rabat, Morocco; 8 Department of Pediatrics, Military Hospital Mohamed V, Hay Riad Rabat, Morocco; 9 Infectious Diseases, Clinical Immunology and Allergy Division, Department of Pediatrics, Cerrahpasa Medical School, Istanbul University, Cerrahpasa, Istanbul, Turkey; 10 Department of Pediatric Infectious Diseases and Immunology, Bakirkoy Maternity and Children's State Hospital, Istanbul, Turkey; 11 Dr. Sami Ulus Children's Health and Diseases Training and Research Center, Ankara, Turkey; 12 Department of Pediatric Infectious Diseases and Clinical Immunology, Istanbul University Faculty of Medicine, Istanbul, Turkey; 13 Department of Pediatrics, Ege University Medical School, Izmir, Turkey; 14 Division of Infectious Diseases and Clinical Immunology, National Research Institute of Tuberculosis and Lung Diseases, Shahid Beheshti University of Medical Sciences, Teheran, Iran; 15 Pediatric Respiratory Disease Research Center, NRITLD, Shahid Beheshti University of Medical Sciences, Teheran, Iran; 16 Pediatric Immunology-Hematology Unit, Necker Hospital, Paris, France; Fundació Institut Germans Trias i Pujol; Universitat Autònoma de Barcelona CibeRES, Spain

## Abstract

**Background and Objectives:**

In the last decade, autosomal recessive IL-12Rβ1 deficiency has been diagnosed in four children with severe tuberculosis from three unrelated families from Morocco, Spain, and Turkey, providing proof-of-principle that tuberculosis in otherwise healthy children may result from single-gene inborn errors of immunity. We aimed to estimate the fraction of children developing severe tuberculosis due to IL-12Rβ1 deficiency in areas endemic for tuberculosis and where parental consanguinity is common.

**Methods and Principal Findings:**

We searched for *IL12RB1* mutations in a series of 50 children from Iran, Morocco, and Turkey. All children had established severe pulmonary and/or disseminated tuberculosis requiring hospitalization and were otherwise normally resistant to weakly virulent BCG vaccines and environmental mycobacteria. In one child from Iran and another from Morocco, homozygosity for loss-of-function *IL12RB1* alleles was documented, resulting in complete IL-12Rβ1 deficiency. Despite the small sample studied, our findings suggest that IL-12Rβ1 deficiency is not a very rare cause of pediatric tuberculosis in these countries, where it should be considered in selected children with severe disease.

**Significance:**

This finding may have important medical implications, as recombinant IFN-γ is an effective treatment for mycobacterial infections in IL-12Rβ1-deficient patients. It also provides additional support for the view that severe tuberculosis in childhood may result from a collection of single-gene inborn errors of immunity.

## Introduction

*“The occurrence of tuberculosis in families led to the view that it was an inherited disease. The demonstration of a characteristic bacterium by Koch in 1882 disposed of this view.”* Theobald Smith [1]

Tuberculosis (TB) is typically caused by *Mycobacterium tuberculosis* (Mtb) and has probably claimed more lives than any other single infectious disease in human history. It continues to be responsible for almost two million deaths each year [2]. Two main forms of clinical disease have been historically observed in endemic areas, at least until the advent of the first antibiotics, and the further blurring of the overall picture that occurred with the HIV pandemic. The disseminated form in children is acute and results from the hematogenous spread of Mtb during primary infection, whereas the chronic pulmonary form in adults, results from the reactivation of latent Mtb infection [3], [4].

A few decades after the discovery of Mtb by Robert Koch in 1882, it became apparent that most people in endemic areas were infected with this bacterium but remained asymptomatic. These findings, obtained at the turn of the twentieth century, were based on both hypersensitivity to subcutaneously [5] and intradermally (Mantoux) administered tuberculin [6] and the growth of Mtb from the lungs of patients dying from other causes [7]. Indeed, only a small fraction of individuals infected with Mtb develop clinical TB in their lifetime. Mtb remains latent in most infected individuals. One century later, the phenomenon of latency, and reactivation thereof, remains largely unexplained, implying that the pathogenesis of adult TB itself is unclear. This is also true for pediatric TB, which occurs in about 5% of infected children during primary infection.

In line with a long tradition of thought, we hypothesize that TB is not only an infectious disease, but also a *bona fide* genetic disorder; more specifically, we hypothesize that TB results from inborn errors of immunity [3], [8]–[12]. The genetic theory of TB, which predates the discovery of Mtb [1], [13], was supported from the 1910s onwards by genetic epidemiology surveys, initially based on correlation studies [14], [15] and twin studies [16]. Studies in the mouse model conducted from the 1920s onwards also provided strong support for the genetic theory of TB [17]. Finally, over the last decade, adult TB susceptibility chromosomal regions were mapped by candidate gene and genome-wide linkage and association studies [3], [8]–[12], [18]–[20].

The human molecular genetic dissection of pediatric TB was facilitated from 1996 onwards by the dissection of genetic etiologies of the syndrome of Mendelian susceptibility to mycobacterial disease (MSMD), which is characterized by clinical disease caused by weakly virulent mycobacteria, such as BCG vaccines and environmental mycobacteria (EM), in otherwise healthy children who are normally resistant to most other infectious agents [8], [21]. In the last 12 years, as many as 15 disorders have been discovered, involving eight genes that control the IL-12-IFN-γ circuit [21]–[24].

These studies paved the way for the identification of the first children with Mendelian predispositions to *bona fide* TB: autosomal recessive IFN-γR1 deficiency in a child not vaccinated with BCG, autosomal recessive IL-12p40 deficiency in a child who also had disseminated BCG disease (known as BCG-osis), XR-MSMD1 (NEMO) in a child without BCG vaccination [21] and XR-MSMD2 (CYBB) in a child not vaccinated with BCG [23]. More convincingly, in three unrelated families from Morocco, Spain, and Turkey, children bearing two loss-of-function alleles of *IL12RB1* were found to suffer from severe TB in the absence of any signs of MSMD, despite vaccination with BCG and clear exposure to EM [25]–[27]. The proband in the Moroccan family suffered from BCG-osis [25], leading to the investigation of a sibling with TB, whereas the other two families had no family history of MSMD [26], [27]. Indeed, IL-12Rβ1 deficiency has been shown to display incomplete clinical penetrance for the case-definition phenotype of MSMD [28], [29].

Based on these studies, the proportion of children with disseminated TB due to monogenic predisposition in endemic areas was proposed, by purely theoretical calculations, to be far from negligible (3% to 30%) [3]. These data and calculations raised two key, general questions: What proportion of children with disseminated TB have a predisposition conferred by single-gene variations? And what are these inborn errors of immunity? As a first approach to tackling this fundamental problem, we attempted to estimate the proportion of children with severe TB due to autosomal recessive IL-12Rβ1 deficiency in three countries endemic for TB, where HIV infection is infrequent, and where consanguineous marriages are common, including Iran, Morocco, and Turkey.

## Results

**Figure 1:**
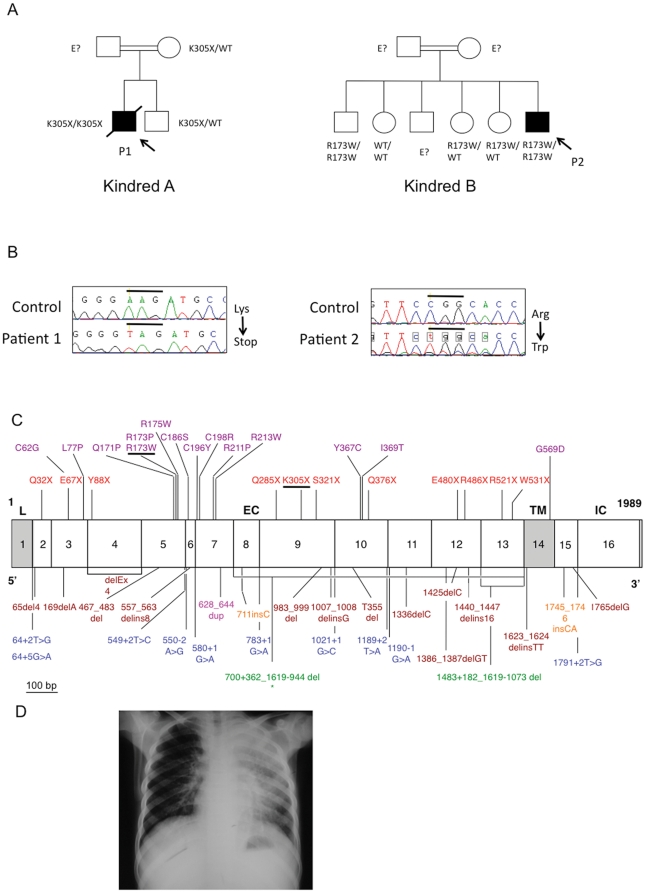
Mendelian mutations in *IL12RB1* leading to severe tuberculosis in two kindreds. **A**. Pedigree of the two families (A and B) with IL-12Rβ1 deficiency. Each generation is designated by a roman numeral (I–II), and each individual by an Arabic numeral. The double lines connecting the parents indicate consanguinity. The probands are indicated by an arrow, with black indicating *Mycobacterium tuberculosis* disease status. Individuals whose genetic status could not be evaluated are indicated by the symbol “E?”. **B**. Electrophoregram showing the genomic sequences of exons 9 and 5 in patients 1 and 2, respectively, compared with a control sequence. **C**. Schematic diagram of the coding region of the IL-12Rβ1 chain containing 17 coding exons and encoding a 662-amino acid protein with a leader sequence (exon1, L), extracellular domain (exons 2 to 13, EC), transmembrane domain (exon 14, TM) and an intracellular cytoplasmic domain (exons 15 to 17, IC). Published and unpublished mutations are indicated as follows: missense mutations are shown in purple, nonsense mutations are shown in red and complex mutations are shown in brown. Splicing mutations are shown in blue, large deletions are shown in green, insertions are shown in orange, and duplication is shown in magenta. * The 700+362_1619-944del mutation is the only mutation resulting in at the expression of a protein at the cell surface. Mutations of P1 (K305X) and P2 (R173W) are underlined. **D**. Chest X ray of patient 1 showing the localization of the disease.

In 48 patients, no rare variations were found. However, we identified two patients carrying homozygous mutations in *IL12RB1*: a mutation in exon 9 in Moroccan P1, leading to the introduction of a stop codon at position 305 (K305X), and a mutation in exon 5 in Iranian P2, leading to the replacement of an arginine residue with a tryptophan residue at position 173 (R173W)(figure 1). These mutations were previously shown not to be polymorphisms by sequencing control samples from various ethnic groups [28], [29] and had been further shown to be loss-of-expression and loss-of-function in other patients with MSMD [28], [29]. Both mutations lead to complete IL-12Rβ1 deficiency and render the patient's cells completely unresponsive to IL-12. These two patients came from unrelated families, each of which was consanguineous, originating from and living in Morocco (P1) and Iran (P2).

P1 had been vaccinated with BCG at birth, with no adverse reaction. He developed severe pulmonary TB at 13 years of age, leading to his death after three months despite treatment with rifampin, isoniazid, pyrazinamide and streptomycin. The presence of Mtb was confirmed bacteriologically by culture from sputum. The patient was the only member of this family homozygous for the mutant allele of *IL12RB1* (his older brother is heterozygous) (Figure 1).

P2 was diagnosed with severe pulmonary tuberculosis at seven months of age, which was bacteriologically confirmed (from gastric lavage), and treated with isoniazid and rifampin (Figure 1). A paraspinal abcess due to Mtb was diagnosed at six years of age, and was treated by surgical resection and antimycobacterial drugs (isoniazid, rifampin and clarithromycin). P2 also had cutaneous leishmaniasis at the age of five years, successfully treated with Glucantime (meglumine antimoniate) for 20 days. An older brother was found to be homozygous for the mutant allele of *IL12RB1*. This brother had been vaccinated with BCG, with no adverse effect, and developed cutaneous leishmaniasis at the age of seven years and scrofuloderma of the neck at the age of 12 years, which was successfully treated with antimycobacterial drugs (rifampin and isoniazid supplemented with streptomycin for the first two months). Unfortunately, no pathological and microbiological investigations were carried out to ascertain the probable Mtb-linked etiology of scrofuloderma. Another IL-12Rβ1-deficient patient with visceral leishmaniasis has previously been described [30]. All the children of this Iranian family, including the two IL-12Rβ1-deficient siblings, were vaccinated with BCG at birth, with no adverse reaction (Figure 1). However, none of the children developed clinical disease caused by BCG or environmental mycobacteria. Thus, in summary, P1 and P2 presented a phenotype of severe TB in childhood, without MSMD. The brother of P2 also probably suffered from TB. There was no history of MSMD in either family. All index cases were investigated based on the sole criterion of being children admitted for severe TB to university hospitals in Morocco, Turkey, and Iran.

## Discussion

This study shows for the first time, at the population level, that in at least two countries with a high rate of consanguineous marriages (Morocco and Iran) severe TB may result from autosomal recessive IL-12Rβ1 deficiency, in at least some children. We investigated 50 patients, two of whom were found to carry homozygous loss-of-function mutations in *IL12RB1*, giving an overall estimated prevalence of 4%. Given the small size of the sample, this estimate is only a rough one; however, it is quite remarkable that we were able to detect two IL-12Rβ1-deficient patients in a sample of only 50 children. It is difficult to assess the actual prevalence of IL-12Rβ1 deficiency as a genetic etiology of pediatric TB in Morocco (1 in 35 samples); however, P1 is the second child to be diagnosed with TB and IL-12Rβ1 deficiency in this country [25]. P2 is the first IL-12Rβ1-deficient child with TB diagnosed in Iran; however, this is probably not unique, as a young adult patient with TB and IL-12Rβ1 deficiency has also recently been identified in this country [31]. Finally, although we found no IL-12Rβ1-deficient Turkish children with TB in this study, we did identify one such child in a previous study [27]. Overall, IL-12Rβ1 deficiency does not seem to be an exceedingly rare genetic etiology of TB, at least in children from Morocco, Turkey, and Iran. We now need to test more children from these countries, as well as children with severe TB from other regions of the world where parental consanguinity is less common. Our previous identification of two Spanish siblings with TB and IL-12Rβ1 deficiency, born to non-consanguineous parents, suggests that there may be other cases around the world [26]. Overall, our study suggests that IL-12Rβ1 deficiency should be considered in selected children with severe TB, at least in areas with a high prevalence of parental consanguinity. This is important clinically, not only for genetic counseling, but also because recombinant IFN-γ is an effective treatment for mycobacterial disease in patients with IL-12Rβ1 deficiency displaying impaired IFN-γ production [21], [32], [33].

In this study, we investigated only one gene, *IL12RB1*, because mutations in this gene had previously been found in three unrelated children with TB [25]–[27]. We focused on its coding region, yet there may be children carrying non-coding and TB-causing mutations in *IL-12RB1*. Moreover, other genes are known to be associated with MSMD and even with childhood TB. Indeed, one child with partial recessive IFN-γR1 deficiency, and others with IL-12p40 deficiency, XR-MSMD1 (NEMO deficiency) and XR-MSMD2 (CYBB deficiency) associated with clinical TB have been identified [21], [23]. Mutations in these genes may be responsible for TB in other children, particularly if the morbid alleles concerned display incomplete penetrance for the case-definition phenotype of MSMD, such as *IL12B* and *IL12RB1*. Mutations in other genes possibly but not necessarily related to IFN-γ-mediated immunity, may also be involved. This study adds weight to the hypothesis that severe TB may be attributable to a collection of single-gene mutations, in at least some children. It is not surprising that recessive traits are frequently involved in countries in which the rate of consanguinity is as high as 20% (Morocco and Turkey) or 38% (Iran). However, in our recent large series of 102 families with an IL-12Rβ1-deficient proband with MSMD [29], only 58 families (57%) were clearly consanguineous and 14 probands were compound heterozygous, indicating that complete IL-12Rβ1 deficiency may also occur in non-consanguineous families. Dominant traits may also be involved, consistent with the identification of various dominant MSMD-causing mutations in *IFNGR1*, *IFNGR2*, and *STAT1* [34]–[37]. We are currently trying to improve our estimate of the percentage of children suffering from genetically determined severe TB by collecting more samples from these three populations (Iran, Morocco and Turkey). We intend to use a genome-wide hypothesis-generating approach, sequencing the ‘whole exome’ and ‘whole genome’ of children with TB enrolled in this study [38]–[40]. The genetic dissection of pediatric TB should have a major impact on our understanding of the pathogenesis of TB and may lead to new therapeutic interventions based on a rational understanding of the pathogenesis.

## Methods

We investigated the *IL12RB1* gene in 50 children from three countries in which tuberculosis is endemic: 11 children from Turkey, 4 from Iran, and 35 from Morocco. These countries were selected because they have a high prevalence of consanguineous marriages (around 20% in Morocco and Turkey [41], [42] and 38% in Iran [43]), a very low prevalence of HIV (less than 0.1% in Morocco and Turkey and less than 0.15% in Iran) (WHO 2010), a high-quality pediatric care and microbiological diagnosis and a high incidence of TB (25–30 per 100,000 persons/year in Turkey and Iran, and 98 per 100,000 persons/year in Morocco) (WHO 2010). This incidence is slightly higher in urban areas such as 40 per 100,000 persons/year in Istanbul and 122 per 100,000 persons/year in Casablanca. In addition, the Arabs and Berbers in Morocco, the Turks in Turkey, and the Persians in Iran form distinctly different ethnic groups. Our study was conducted in accordance with the Helsinki Declaration, with written informed consent obtained from each patient or the patient's family. The IRB was obtained and approved for this study in France (CPP committee C1016) by INSERM and INSERM serves as IRB of record for all institutions/hospitals. The patients herein reported were not described in our recent review of 141 patients with IL-12Rβ1 deficiency, who were selected on the basis of their personal or familial phenotype of MSMD [29]. All the patients studied displayed confirmed severe tuberculosis requiring hospitalisation during childhood, had no classical primary immunodeficiency and were not infected with HIV. All the patients that (i) fulfilled the inclusion criteria, (ii) whose parents agreed to participate and (iii) for whom biological material was recovered, have been analysed. This represents a total of 50 patients. Most (88%) had been vaccinated with BCG, with no adverse effects. The clinical presentations of TB recorded included miliary TB (n = 21), TB meningitis (n = 12), peripheral TB adenitis (n = 9) (i.e. infection of peripheral lymph nodes), mediastinal TB adenitis (n = 14) (i.e. infection of mediastinal lymph nodes), pulmonary TB (n = 10), urinary TB (n = 1) and TB osteitis (n = 2). Most (35) patients presented TB at a single location (Table 1). For all 50 patients, the coding and flanking intron sequences of *IL12RB1* were amplified [44] and sequenced with an ABI 3730x sequencer (Applied Biosystems).

**Table 1:** Table indicating the clinical presentations of TB recorded in the fifty patients.

N	Sex	Age	Origin	BCG	Miliary TB	TB Meningitis	Peripheral TB	Mediastinal TB	TB osteitis	Pulmonary TB	Urinary TB
1	F	0.5	Morocco	yes	yes			yes			
2	M	0.25	Morocco	yes	yes						
3	M	1.5	Morocco	yes	yes						
4	F	3	Morocco	yes		yes					
5	F	0.83	Morocco	yes	yes		yes	yes			
6	M	6	Morocco	yes	yes	yes					
7	F	7	Morocco	yes					yes		
8	F	1.16	Morocco	yes	yes	yes					
9	M	9	Morocco	yes			yes	yes			
10	M	11	Morocco	yes				yes			
11	F	3	Morocco	yes			yes			yes	
12	M	10	Morocco	yes				yes			
13	F	3	Morocco	yes	yes		yes				
14	F	12	Morocco	yes				yes			
**15**	**M**	**13**	**Morocco**	**yes**						yes	
16	M	5	Morocco	yes				yes			
17	F	10	Morocco	yes				yes			
18	F	11	Morocco	yes		yes					
19	M	10	Morocco	yes	yes						
20	F	4	Morocco	yes	yes						
21	M	4	Morocco	yes		yes					
22	M	9	Morocco	yes		yes					
23	F	8	Morocco	yes	yes			yes			
24	F	4	Morocco	yes	yes						
25	F	1.1	Morocco	yes	yes						
26	M	2	Morocco	yes			yes	yes		yes	
27	M	0.5	Morocco	yes	yes						
28	M	10	Morocco	yes			yes	yes		yes	
29	M	3.5	Morocco	yes		yes					
30	F	2	Morocco	yes	yes						
31	M	1.2	Morocco	yes	yes						
32	F	6	Morocco	yes	yes						
33	M	4	Morocco	yes	yes						
34	F	5	Morocco	yes	yes		yes				
35	M	9	Morocco	yes	yes						
36	F	1.8	Turkey	no		yes					
37	F	0.45	Turkey	yes		yes					
38	F	15	Turkey	yes		yes					
39	M	4	Turkey	no				yes			
40	M	3	Turkey	no		yes					
41	F	15	Turkey	yes			yes	yes			
42	M	14	Turkey	no	yes						
43	F	0.75	Turkey	no							yes
44	M	6	Turkey	yes					yes		
45	F	14	Turkey	yes				yes		yes	
46	F	10	Turkey	no	yes	yes				yes	
**47**	**M**	**0.58**	**Iran**	**yes**			yes			yes	
48	F	12	Iran	yes						yes	
49	F	15	Iran	yes						yes	
50	F	14	Iran	yes						yes	

The two patients presenting with IL-12Rβ1 deficiency are in bold. N means number and age is indicated in years.
